# Effect of Zn doping on phase transition and electronic structures of Heusler-type Pd_2_Cr-based alloys: from normal to all-d-metal Heusler†

**DOI:** 10.1039/d0ra02951c

**Published:** 2020-05-07

**Authors:** Xiaotian Wang, Mengxin Wu, Tie Yang, Rabah Khenata

**Affiliations:** School of Physical Science and Technology, Southwest University Chongqing 400715 China; Laboratoire de Physique Quantique de la Matière et de la Modélisation Mathématique (LPQ3M), Université de Mascara Mascara 29000 Algeria khenata_rabah@yahoo.fr

## Abstract

Based on first-principles calculations, for Heusler alloys Pd_2_CrZ (Z = Al, Ga, In, Tl, Si, Sn, P, As, Sb, Bi, Se, Te, Zn), the effect of Zn doping on their phase transition and electronic structure has been studied in this work. These alloys can be divided into two classes: (i) all-d-metal Heusler Pd_2_CrZn and (ii) other normal Heusler alloys Pd_2_CrZ (Z = Al, Ga, In, Tl, Si, Sn, P, As, Sb, Bi, Se, Te). For all-d-metal Heusler alloy Pd_2_CrZn, transition metal element Zn behaves like a main group element due to its full 3d occupied state, and therefore the Zn atoms tend to occupy Wyckoff sites D (0.75, 0.75, 0.75) instead of replacing Pd atoms at A sites (0, 0, 0). The stable tetragonal L1_0_ state is obtained *via* tetragonal deformation and the L1_0_ stable state can be tuned by the uniform strain. The stability of the tetragonal state is analyzed and proved *via* calculation of the density of states (DOSs) and the phonon spectrum. For the series of normal Heusler alloys Pd_2_CrZ, doping with Zn atoms can induce or strengthen the martensitic transformation, or regulate the large *c*/*a* ratios to a more reasonable range. It is hoped that this work can provide some guidance for further studies of the relationship between all-d-metal and normal Heusler alloys in the future.

## Abstract 2

By first-principles calculations, for Heusler alloys Pd_2_CrZ (Z = Al, Ga, In, Tl, Si, Sn, P, As, Sb, Bi, Se, Te, Zn), the effect of Zn doping on their phase transition and electronic structure has been studied in this work.

## Graphical abstract

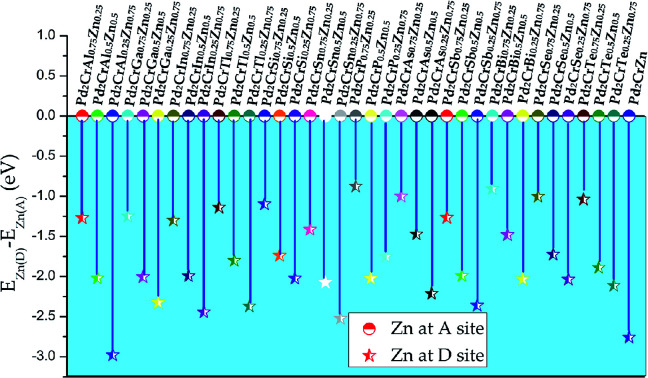

## Introduction

Magnetic phase transition materials^1^ are novel smart materials that can sense and respond to environmental excitation and have been widely used in magnetic drives, magnetic sensors, magnetic storage, solid-state refrigeration, thermoelectric conversion and so on.^2,3^ Searching for new magnetic phase transition materials is an essential study in the intelligent materials field. Magnetic shape memory alloys (MSMAs), one of the magnetic phase transition materials, play a pivotal role in magnetic actuation, magnetic refrigeration and the spintronics field.^4,5^ Therefore, it is important to design and synthesize novel MSMAs.

Heusler alloys^6^ are the largest family in MSMAs, and their excellent characteristics make them a hot topic of study in such fields as modern electronic information technology, aerospace, and mechanical electronics. Heusler alloys become a favourable candidate for MSMAs due to their high Curie temperature,^7,8^ tunable electronic structures,^9,10^ adaptable semiconductor lattice constant^11^ and novel magnetic and structural properties.^12–14^ Normally, Heusler alloys have three classic types: half-Heusler alloys (XYZ),^10^ full-Heusler alloys (X_2_YZ),^15,16^ and equiatomic quaternary Heusler alloys (XYMZ).^13,17,18^ Although the Heusler system has been proposed for more than 100 years, this system retains a high interest due to a series of novel physical phenomena and concepts were observed in this old system. A new category of Heusler family, all-d-metals,^19^ consists entirely of transition metal elements have been proposed recently. These alloys exhibit better phase space and versatility than conventional Heusler materials and feature magnetic martensitic transformation, magnetic drive shape memory, large magnetic drive strain, large magnetoresistance, large magnetocaloric effect, and large volume expansion.^19–22^

Since the first MSMA Heusler alloy, Ni–Mn–Ga,^23^ was proposed and studied in depth, other Heusler MSMA alloys have been widely investigated experimentally and theoretically, such as Ni_2_FeGa,^24^ Co–Ni–Ga (Al),^25^ Mn_2_NiGa,^26^ and Fe–Mn–Ga.^27^ Very recently, there have been some studies of all-d-metal Heusler alloys: (i) in the early 1900s, Muldawer *et al.* reported two all-d-metal Heusler alloys, Zn_2_AuAg and Zn_2_CuAu,^28^ with ordered L2_1_ or disordered B_2_ structures. However, the nonmagnetic properties of these alloys limit the scope of application in magnetic materials, this study has spurred interest in all-d-metal Heusler alloys. (ii) Wei *et al.* achieved the multifunctional ferromagnetic shape memory effect in all-d-metal Ni_50−*x*_Co_*x*_Mn_50−*y*_Ti_*y*_ in 2015.^19^ Doping transition group element Ti to the Ni–Mn system can benefit forming the cubic B_2_ Heusler structures and stable the parent phase, further leading to the martensitic transformation. Based on the Ti–Ni–Mn phase, a strong ferromagnetic coupling in the Ni(Co)–Mn–Ti alloy was found *via* Co substitution. (iii) Wei *et al.* then conducted subsequent studies on the martensitic transformation and magnetic strain of the Ni_50−*x*_Co_*x*_Mn_50−*y*_Ti_*y*_ system with large volume changes in 2016.^21^ They found that the martensitic transformation of Ni_50−*x*_Co_*x*_Mn_50−*y*_Ti_*y*_ can occur at a magnetic strain of 6900 ppm and Ni_50−*x*_Co_*x*_Mn_50−*y*_Ti_*y*_ polycrystalline samples exhibit a volume change of 2.54% near the room temperature. (iv) In 2018, Han *et al.* investigated the possible martensitic transformation of a series of all-d-metal equiatomic quaternary Heusler hypothetical alloys, ZnCdTMn (T = Fe, Ru, Os, Rh, Ir, Ni, Pd, Pt), by means of first-principle calculation.^20^ (v) Ni *et al.* studied the magnetic structural transition of Mn_2_Ni_1.5_Ti_0.5_ and Mn_2_Ni_1.25_Co_0.25_Ti_0.5_ and they found that the energy difference between the antiparallel and parallel configurations of the Mn (B) and Mn (D) moments can be increased by Co doping in Mn_2_Ni_1.25_Co_0.25_Ti_0.5_.^29^ According to these previous studies, doping transition metal elements may be a favorable method to obtain all-d-metal Heusler alloys with magnetic phase transitions.

Therefore, in this work, we will study the doping effect of transition metal element Zn on Heusler alloys Pd_2_CrZ, which may enable adjusting their phase transition and other magnetic properties for better applications under different conditions. To our best knowledge, there are no other studies focusing on the effect of Zn doping in Heusler alloys Pd_2_CrZ, and therefore, current work can be seen as a reference for future theoretical and experimental investigations. The reasons we selected Zn element in this manuscript can be shown as follows: (i) currently, 3d metals doping to MSMAs is quite widespread. Among them, Zn has a full d (3d^10^4s^2^) occupied state of the atom, which causes the indirect interaction between Zn and other transition metal atoms in the Heusler alloys due to its low d-state energy, so the Zn atom may be regarded as the same role as a main group element. Some investigations have been carried out and found that Zn doping can improve the phase transition temperature or stability as well as the ordering of the structure;^30–32^ however, a general first principle study to discuss the effect of Zn doping in normal Heusler alloys is still needed. (ii) The second reason is that the price of Zn is relatively lower than other transition metal elements, and thus it will be less costly for industrial applications.

## Materials and methods

All the electronic structures have been computed *via* the density functional theory^33^ which is realized in the VASP code^34^ in this work. The exchange and related functions are described by Perdew–Burke–Ernzerhof (PBE)^35^ for the parameterization of GGA.^36^ And the projector augmented wave (PAW)^37^ was chosen to handle the interaction between the valence electrons and ion cores. In all situations, the plane-wave basis set cutoff is set as 450 eV and the Monkhors–Pack special *k*-point mesh is fixed to 12 × 12 × 12 in all Brillouin zone integration whether in XA- or L2_1_-type Heusler alloys. The unit cell was optimized until the force and total energy to less than 0.005 eV Å^−1^ and 0.0000001 eV, respectively. The phonon energy calculation of Pd_2_CrZ was performed in NanoAcademic Device Calculator (Nanodcal) code.^38^

**Fig. 1:**
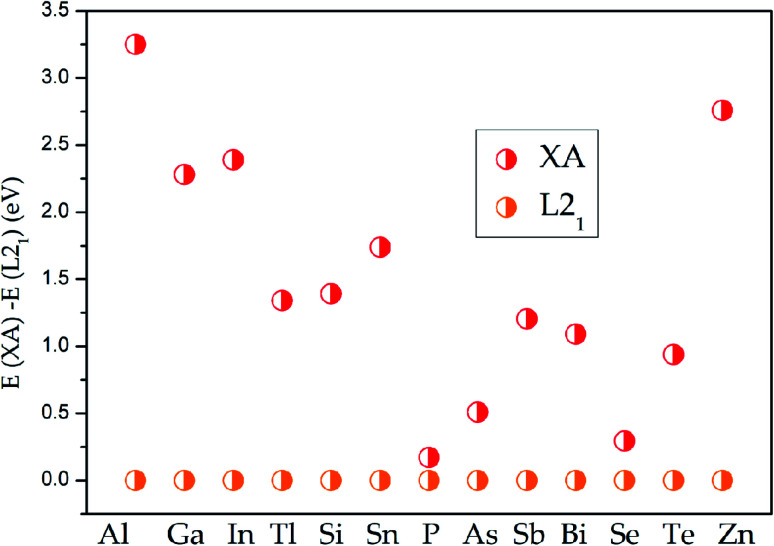
Energy differences between XA and L2_1_ states for all the Pd_2_CrZ alloys (Z = Al, Ga, In, Tl, Si, Sn, P, As, Sb, Bi, Se, Te, Zn).

**Fig. 2:**
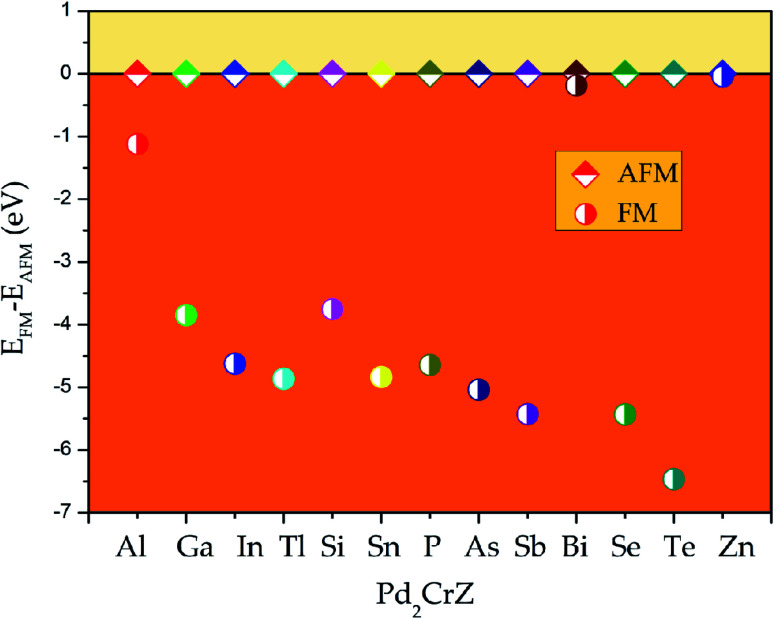
Δ*E* = *E*_FM_ − *E*_AFM_ per formula unit as a function of Z (Z = Al, Ga, In, Tl, Si, Sn, P, As, Sb, Bi, Se, Te, Zn) for all the Pd_2_CrZ (Z = Al, Ga, In, Tl, Si, Sn, P, As, Sb, Bi, Se, Te, Zn) with L2_1_ type structure.

To determine the ground state of Pd_2_CrZ (Z = Al, Ga, In, Tl, Si, Sn, P, As, Sb, Bi, Se, Te, Zn), the crystals of full Heusler-type alloys Pd_2_CrZ were optimized. The site-preference rule (SPR)^39^ is usually employed to determine the possible atomic preference of full-Heusler alloys X_2_YZ. Normally, when X atoms have more valence electrons than Y, X atoms tend to locate at the Wyckoff A and C sites, the Y atoms tend to locate at B sites, as well as the main group element Z occupies the D sites, forming the L2_1_-type Heusler alloys. The other form is the XA-type Heusler alloys, in which the valence electrons in X are less than Y atoms, so the A and B sites are occupied by X atoms. As shown in Fig. S1,† the crystal structures of XA- and L2_1_-type of Pd_2_CrZ are plotted. From Fig. 1, the energy differences between XA and L2_1_ structures were given. From it, one can see that the L2_1_ is the stable crystal structure due to its lower energy than the XA-type. Based on the L2_1_ phase, the magnetic states, including ferromagnetic and antiferromagnetic (AFM), for Pd_2_CrZ are also taken into consideration. For the AFM state, the antiparallel coupled spin moments between the Mn and Pd atoms are added. One can clearly see that the energy differences (*E*_FM_ − *E*_AFM_) in Fig. 2 of all the Heusler alloys Pd_2_CrZ are negative. That is, the FM state of all Pd_2_CrZ Heusler alloys is more stable than the AFM state.

## Results and discussion

### The phase transition and electronic structures of all-d-metal Pd_2_CrZn

**Fig. 3:**
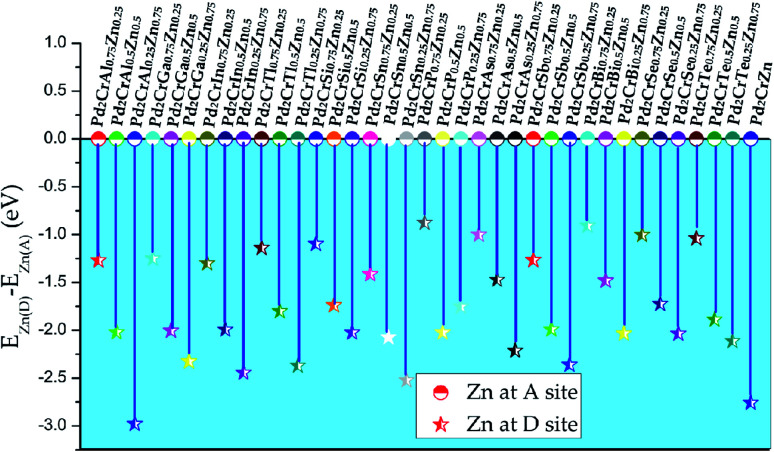
The total energy differences between the alloys with Zn doping atoms at D site and at A site for all the Pd_2_CrZ alloys (Z = Al, Ga, In, Tl, Si, Sn, P, As, Sb, Bi, Se, Te, Zn).

Based on above discussion, one can see that Pd_2_CrZ prefers to exhibit the L2_1_ structure, that is, these main group elements Z locate at Wyckoff site D (0.75, 0.75, 0.75). However, when the Z elements were substituted by the transition metal element Zn, the situation is complicated. We selected Pd_2_CrAl_0.75_Zn_0.25_ as an example: there were two possible formed structures: one structure is that Zn enters the D site where it chemically replaces Al (see Fig. S2(b)†); the other structure is that Zn occupies the Pd (A or C) site and drives some Pd atoms to the D site (see Fig. S2(a)†). The second structure is based on the well-known SPR,^39,40^ which the A or C sites prefer to be occupied by the transition metal elements with more valence electrons, here is the Zn atom. According to the energy differences (*E*_(Zn)D_ − *E*_(Zn)A_) between the D-site doping and A-site doping for all the Pd_2_CrZ_*x*_Zn_1−*x*_ alloys (*x* = 0.25, 0.5, 0.75) in Fig. 3, one can see that D-site Zn doping is preferred based on all the negative energy difference. Therefore, it can be inferred that Zn substitution Z does not change the atomic ordering sites (L2_1_ structure) of Pd and Cr atoms in Pd_2_CrZ_*x*_Zn_1−*x*_, which can be explained by the filled 3d shell of Zn.^30–32^ This is different from some other 3d transition metals such as Co. For instance, in Co-doped Mn_2_NiGa, Co enters the Mn (A) site and drives Mn to the Ga (D) site.^41^

**Fig. 4:**
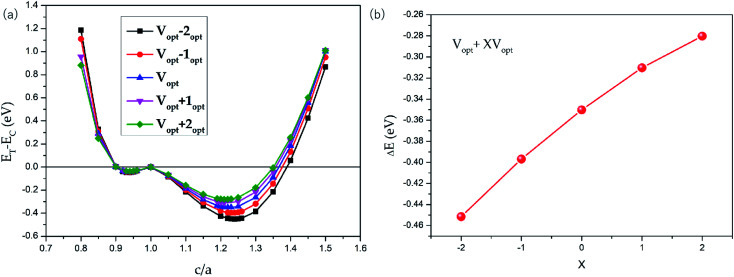
(a) Total energies as functions of the *c*/*a* ratio for tetragonal Pd_2_CrZn with contraction/expansion of the unit cell volume. The zero point of the total energy was set as the energy of the cubic (*c*/*a* = 1). (b) Δ*E* as functions of the (1 + *X*%) *V*_opt_ (*X* = −2, −1, 0, 1, 2) for Pd_2_CrZn.

The possible L2_1_ and L1_0_ phase competition for all-d-metal Pd_2_CrZn alloys have been carried out. The tetragonal strain was added to the L2_1_-type cubic crystal to obtain the L1_0_ tetragonal structure (see Fig. S3†). As shown in Fig. 4, *via* the tetragonal deformation, one can see that Pd_2_CrZn has two local minimums of energy: one at *c*/*a* < 1 and the other at *c*/*a* > 1, indicating the stable tetragonal states of Pd_2_CrZ than cubic states. The local minimum of shallow energy at *c*/*a* = ∼0.94 indicates the existence of the metastable phase here. The deeper energy local minimum occurs at *c*/*a* = ∼1.22 is about 0.35 eV energy difference. Moreover, changing of the uniform strain can tune the Δ*E*_M_. In Fig. 4, we also plotted some phase transitions in different volumes, *V*_opt_ + *X*% *V*_opt_ (*X* = −2, −1, 0, 1, 2). One can see that the absolute values of energy difference (*E*_T_ − *E*_C_) gradually increases from approximately 0.28 eV to 0.45 eV with X varying from +2 to −2, reflecting that the tetragonal L1_0_ states become more and more stable with the volume reduction. In addition, the tetragonal L1_0_ state occurs at around *c*/*a* = 1.2–1.3, similar to the normal tetragonal Heusler alloy Ni_2_MnGa.^23^

**Fig. 5:**
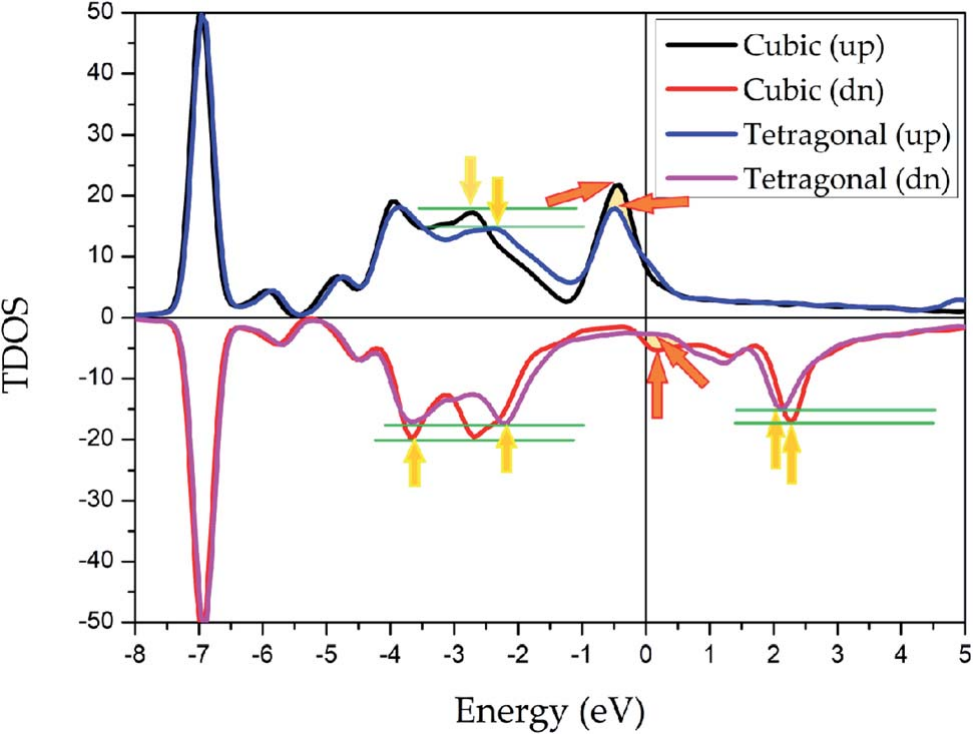
Total density of states (TDOSs) of Pd_2_CrZn alloy in cubic and tetragonal phases.

**Fig. 6:**
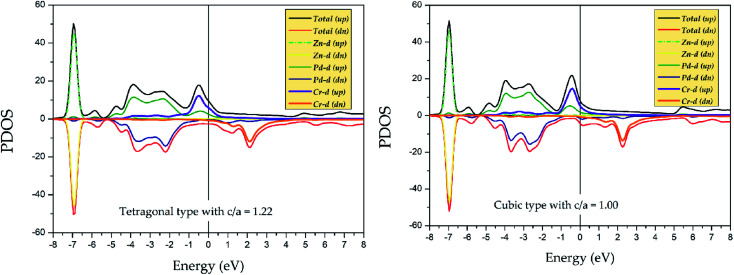
Partial density of states (PDOSs) of Pd_2_CrZn alloy in tetragonal (*c*/*a* = 1.22) and cubic phases.

To explore the stability of tetragonal L1_0_ state of Pd_2_CrZ, we utilized the density of states (DOSs), including the total density of states (TDOSs) and partial density of states (PDOSs), to make a detailed discussion. Pd_2_CrZn alloy was selected as an example and its TDOSs and PDOSs in both cubic and tetragonal states are shown in Fig. 5 and 6. Just considering the TDOSs in or at the vicinity of the Fermi level (*E*_F_), smoother DOSs and lower peaks in the L1_0_-state can be seen as an evidence that the tetragonal state^42^ is more stable than the cubic state of Pd_2_CrZn. Further, whether in tetragonal or cubic phases, the d states of Zn mostly locate in a lower DOS region around −7 eV. Therefore, differ from other transition metal atoms, such as Mn and Co, in Heusler alloys, the direct hybridization between the d electrons of the Zn and Pd (Cr) atoms can be ignored. This also indicates that the Zn atoms behave as the main group elements near *E*_F_. Meanwhile, the DOSs of Pd atoms are nearly symmetrical in both spin channels for the L2_1_ and L1_0_ states. Hence, the magnetic moments of Pd and Zn atoms are quite small. The main contribution to total magnetic moments is coming form the Cr atoms due to their strong spin splitting around the *E*_F_. All the possible phase transitions as well as total and atomic magnetic moments of Pd_2_CrZ were also calculated, and the results are shown in Tables S1 and S2.† In addition, during the tetragonal deformation, the *c*/*a* ratios for most of the Pd_2_Cr-based alloys are approximately 1.2–1.3, while a small part exceeded 1.4, such as in the Pd_2_CrSn and Pd_2_CrP alloys.

### Zn doping of Pd_2_Cr-based full-Heusler alloys: from normal to all-d-metal Heusler

**Fig. 7:**
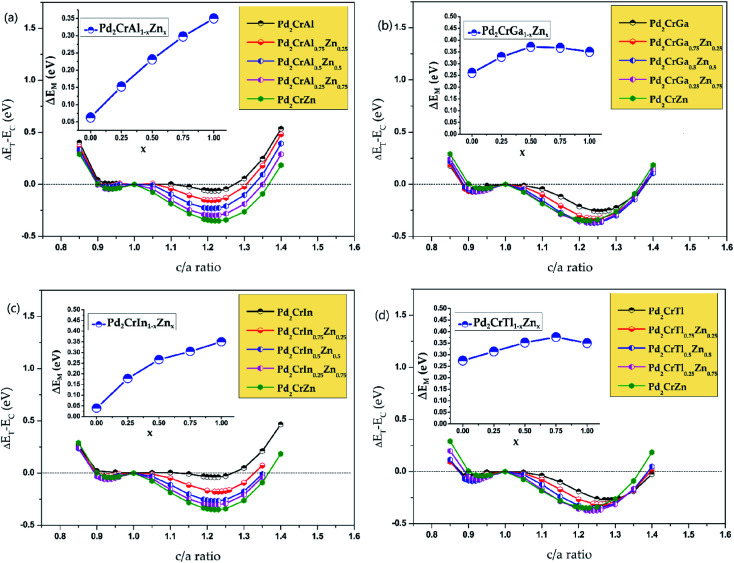
The total energy differences between tetragonal and cubic states as functions of the *c*/*a* ratio of different amount of Zn doping in Pd_2_CrZ (Z = Al, Ga, In, and Tl). The zero point of the total energy was set as the energy of the cubic states (*c*/*a* = 1).

After studying the all-d-metal Heusler alloy Pd_2_CrZn, the normal Heusler alloys Pd_2_CrZ (Z = Al, Ga, In, Tl, Si, Sn, P, As, Sb, Bi, Se, Te) doping with Zn atom will now be further studied. Zn doping with varying degrees of 25%, 50%, 75% and 100% on the Z (D) site are investigated for all the Pd_2_CrZ alloys, and the results are exhibited in Fig. 7 and S4.† There are three situations after Zn doping during the tetragonal deformation. (i) One is the degree of tetragonal transformation is enhanced; that is, the more stable L1_0_ state was found during Zn doping. For Pd_2_CrZ (Z = Al, Ga, In, Tl, Si, As, Te), a more stable state occurred at certain concentrations of Zn doping (see Fig. 7), reflecting that Zn doping in Pd_2_CrZ (Z = Al, Ga, In, Tl, Si, As, Te) can increase the martensitic transformation temperature.^32^ (ii) For some normal Heusler alloys, such as Pd_2_CrZ (Z = Sn, Sb, Bi) (see Fig. S4(b), (e) and (f)†), doping Zn in Pd_2_CrZ (Z = Sn, Sb, Bi) can induce martensitic transformation. (iii) For the Pd_2_CrP and Pd_2_CrSe alloys (see Fig. S4(c) and (g)†), although the Δ*E*_M_ decreases during the Zn doping, the large unreachable *c*/*a* ratios may be regulated to a more reasonable range. In detail, the *c*/*a* ratios of Pd_2_CrP and Pd_2_CrSe are relatively large, approximately 1.5, which may not easy to achieve experimentally. As shown in Fig. S5,† the different degree of Zn doping as functions of the *c*/*a* ratio of Pd_2_CrSe_1−*x*_Zn_*x*_ and Pd_2_CrP_1−*x*_Zn_*x*_ are plotted as examples. From this, it can be seen that doping Zn in Pd_2_CrP and Pd_2_CrSe can gradually adjust the *c*/*a* ratio to a lower value, which may be easy to achieve experimentally.

**Fig. 8:**
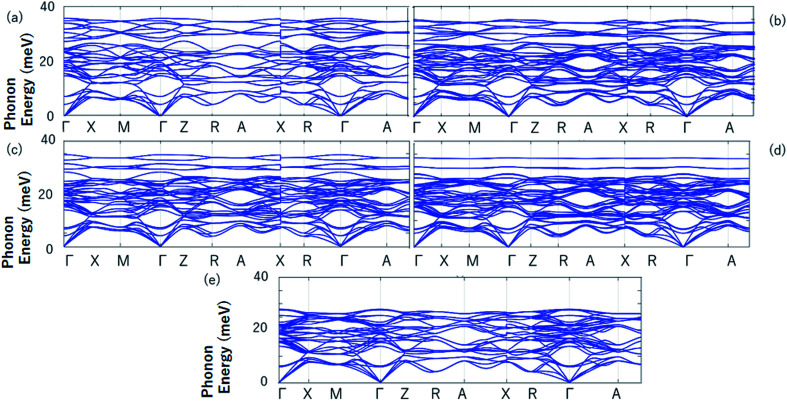
The calculated phonon energies of Pd_2_CrAl (a), Pd_2_CrAl_0.75_Zn_0.25_ (b), Pd_2_CrAl_0.5_Zn_0.5_ (c), Pd_2_CrAl_0.25_Zn_0.75_ (d) and Pd_2_CrZn (e).

Finally, the phonon spectrums have been calculated and the stability of tetragonal Pd_2_Cr-based Heusler alloys with different amounts of Zn doping was examined. Take the Pd_2_CrAl_*x*_Zn_1−*x*_ system (see Fig. S6†) as an example, the absence of a virtual frequency guarantees the stability of the tetragonal state of Pd_2_CrAl with increasing Zn content on the D site (see Fig. 8). Therefore, the tetragonal L1_0_ states of Pd_2_CrAl_*x*_Zn_1−*x*_ are structurally stable. Unfortunately, the possible L2_1_–L1_0_ phase transition of Pd_2_CrZ have not been studied experimentally, and therefore, a comparison between the theoretical and experimental results can not be exhibited in this manuscript. However, the current study can help to understand the physics in all-d-metal alloys and to design new MSMAs in them. Furthermore, we would like point out that Bain paths are a sophisticated way^43^ to investigate the reversible transformation between the L2_1_ and L1_0_ phases during the tetragonal distortion. This method has been widely used to design new MSMAs, some designed MSMAs have been experimentally verified, such as Mn–Ni–Co–Ti system.^22,29^

## Conclusions

In this manuscript, we studied the effect of Zn doping on the phase transition and electronic structures of Heusler-type Pd_2_Cr-based alloys based on spin-polarized first-principles calculations and the following conclusions were obtained:

(i) The L2_1_ and XA competition of full-Heusler Pd_2_CrZ Heusler alloys were examined, indicating that the L2_1_ type is the stable atomic ordering than XA type for Pd_2_CrZ Heusler alloys;

(ii) The FM and AFM magnetic states of the L2_1_ type Pd_2_CrZ Heusler alloys were discussed, and we found that all these alloys are in FM state;

(iii) In Pd_2_CrZ_1−*x*_Zn_*x*_, the Zn atom behaves similarly to the main group element Z, which is usually located at the D position, and the phenomenon of this mechanism is that the full 3d-shell of Zn atom. With the help of the calculated PDOS, in which the d state of Zn is quite far from the *E*_F_. Thus, only the s and p states of Zn are located near the *E*_F_ to hybridize with the transition metal elements Pd and Cr;

(iv) The possible martensitic transition can be found in all-d-metal Pd_2_CrZn alloys, and the austenite-martensitic energy difference Δ*E*_M_ in Pd_2_CrZn can be regulated by uniform strain;

(v) The strong spin splitting of Cr atom around the *E*_F_ makes mainly contribution to the total magnetic moment of Pd_2_CrZn. A lower DOSs around the *E*_F_ was used to explain the stability of the tetragonal state existed in Pd_2_CrZn;

(vi) The martensitic transition and magnetism of Zn-doped Heusler alloys Pd_2_CrZ_1−*x*_Zn_*x*_ (*x* = 0, 0.25, 0.5, 0.75, 1) were investigated, we found the Δ*E*_M_ or *c*/*a* ratio in Pd_2_CrZ_1−*x*_Zn_*x*_ can be tuned on demand with the doping of Zn atoms;

(vii) The calculated phonon spectrums can be seen as a evidence for the stability of the tetragonal state of the Pd_2_CrAl_*x*_Zn_1−*x*_ system;

(viii) Finally, it is hoped that all-d-metal alloys can receive some more attention from researchers; we firmly believe that there may be many other novel physical properties in all-d-metal alloys.

## Conflicts of interest

There are no conflicts to declare.

## Supplementary Material

RA-010-D0RA02951C-s001
