# Supplementary material for: Effect of Zn doping on phase transition and electronic structures of Heusler-type Pd2Cr-based alloys: from normal to all-d-metal Heusler
Source: RSC Adv. 2020 May 7;10(30):17829–35. doi: 10.1039/d0ra02951c (PMC9053613; doi:10.1039/d0ra02951c)
Supplement: RA-010-D0RA02951C-s001 [file RA-010-D0RA02951C-s001.pdf]

This journal is © The Royal Society of Chemistry 2020

Table S1. Total and atomic magnetic moments for cubic L2_1 Pd_2CrZ (Z = Al, Ga, In, Tl, Si, Sn, P, As, Sb, Bi, Se, Te, Zn).

**Compounds** X_2YZ	**Mt** (μ_B/f.u.)	**MCr** (μ_B)	**MZ** (μ_B)	**MPd-1** (μ_B)	**MPd-2** (μ_B)
Pd_2CrAl	3.50	3.25	-0.02	0.13	0.13
Pd_2CrGa	3.53	3.30	-0.04	0.13	0.13
Pd_2CrIn	3.69	3.46	-0.04	0.10	0.10
Pd_2CrTl	3.66	3.51	-0.03	0.09	0.09
Pd_2CrSi	3.33	3.22	-0.04	0.07	0.07
Pd_2CrSn	3.55	3.48	-0.06	0.06	0.06
Pd_2CrP	3.22	3.27	-0.57	0.003	0.003
Pd_2CrAs	3.40	3.41	-0.46	0.018	0.018
Pd_2CrSb	3.56	3.55	-0.04	0.02	0.02
Pd_2CrBi	3.74	3.69	-0.04	0.04	0.04
Pd_2CrSe	3.56	3.58	-0.06	0.02	0.02
Pd_2CrTe	3.71	3.70	-0.05	0.02	0.02
Pd_2CrZn	3.79	3.38	-0.001	0.20	0.203.61

Table S2. Total and atomic magnetic moments, and c/a ratio for tetragonal $L1_0$ Pd_2CrZ (Z = Al, Ga, In, Tl, Si, Sn, P, As, Sb, Bi, Se, Te, Zn).

**Compounds X_2YZ**	**Mt (μ_B/f.u.)**	**MCr (μ_B)**	**MZ (μ_B)**	**MPd-1 (μ_B)**	**MPd-2 (μ_B)**	**c/a**
Pd_2CrAl	3.55	3.23	-0.02	0.17	0.17	1.21
Pd_2CrGa	3.52	3.27	-0.03	0.15	0.15	1.26
Pd_2CrIn	3.82	3.53	-0.01	0.15	0.15	1.22
Pd_2CrTl	3.75	3.50	-0.01	0.13	0.13	1.28
Pd_2CrSi	3.16	3.09	-0.06	0.06	0.06	1.21
Pd_2CrSn	_	_	_	_	_	_
Pd_2CrP	3.02	3.10	0.01	-0.11	-0.11	1.43
Pd_2CrAs	3.14	3.21	-0.1	0.02	0.02	1.43
Pd_2CrSb	3.41	3.46	-0.075	0.009	0.009	1.18
Pd_2CrBi	_	_	_	_	_	_
Pd_2CrSe	3.42	3.47	-0.104	0.02	0.02	1.45
Pd_2CrTe	3.67	3.62	-0.06	0.05	0.05	1.35
Pd_2CrZn	3.49	3.24	-0.009	0.12	0.12	1.22

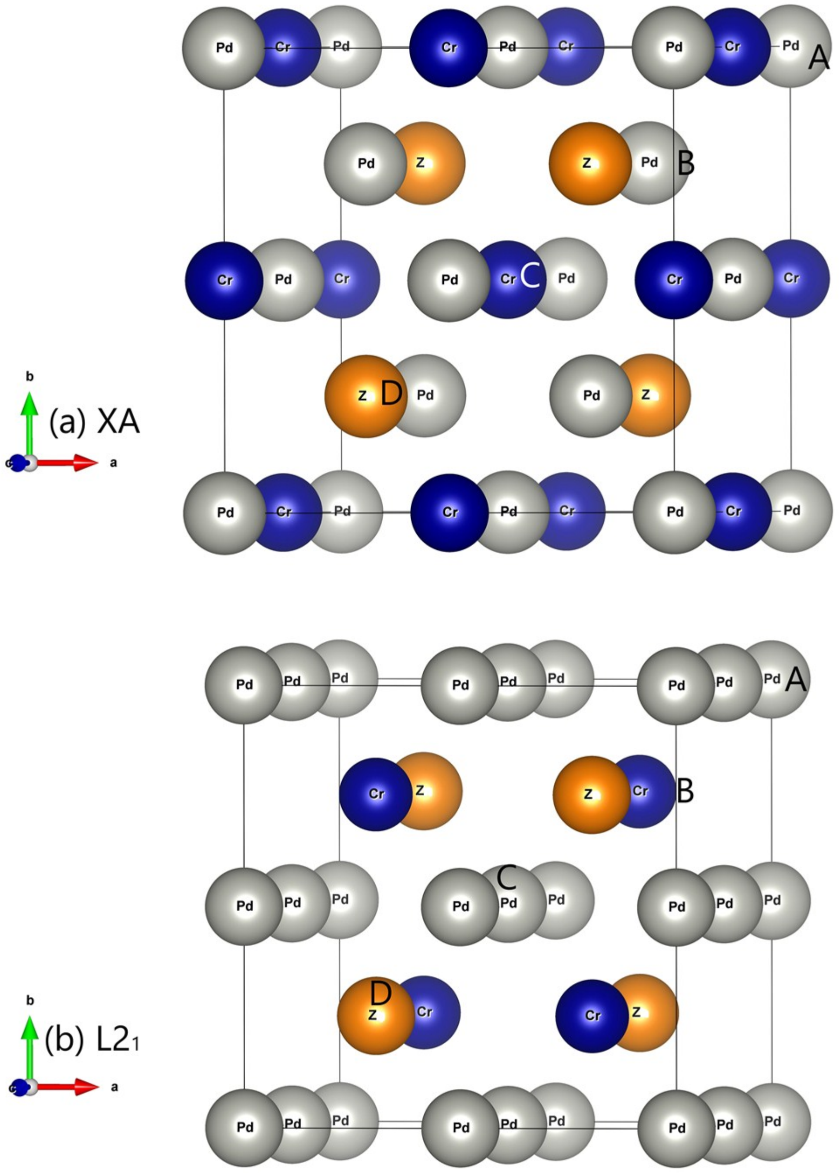

Fig. S1. Crystal structures of XA (a) and $L2_1$ (b)-type Heusler alloys Pd_2CrZ.

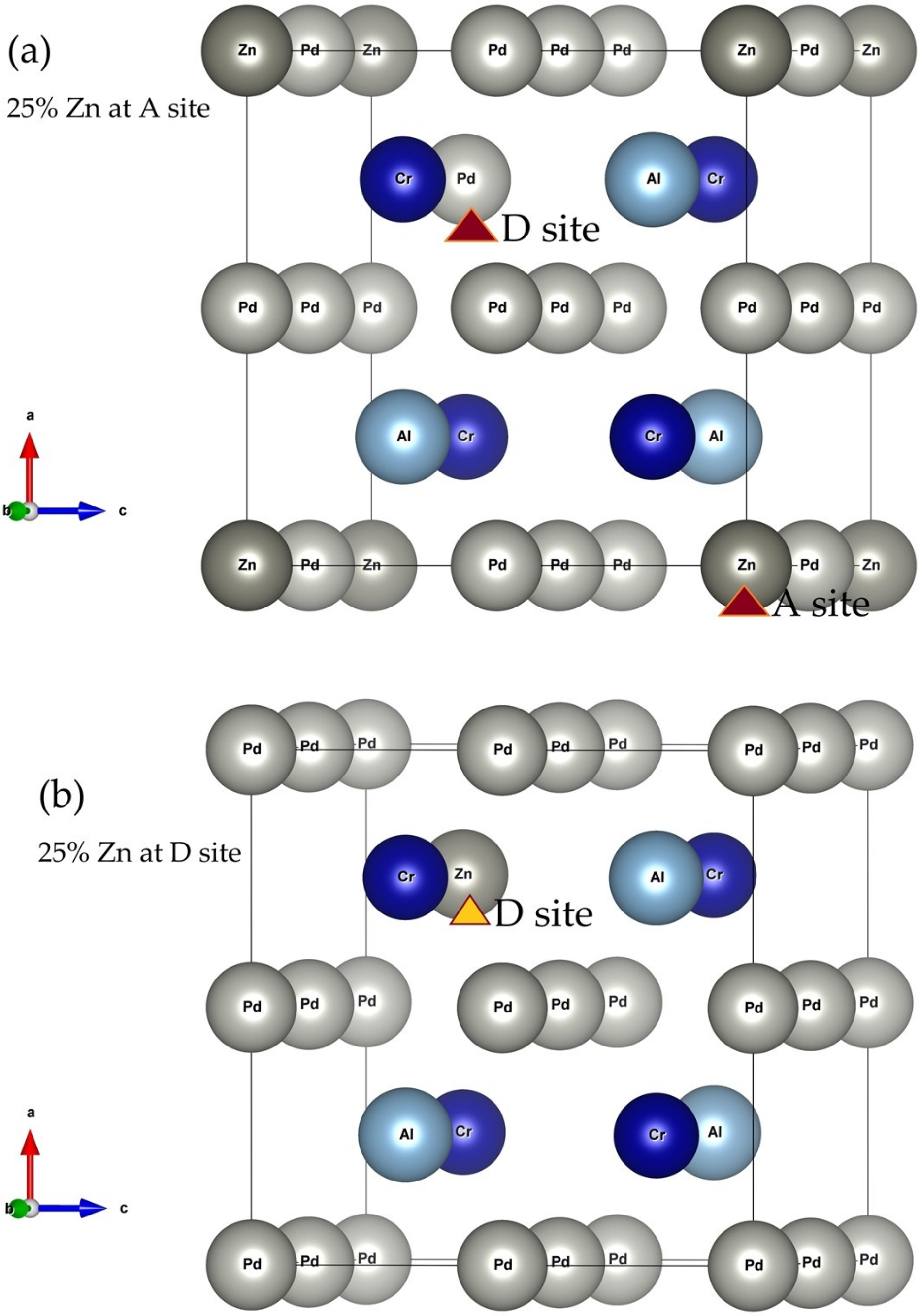

Fig. S2. Crystal structures of Pd_2CrAl doping with 25 % Zn at Wyckoff sites (a) A or (b) D.

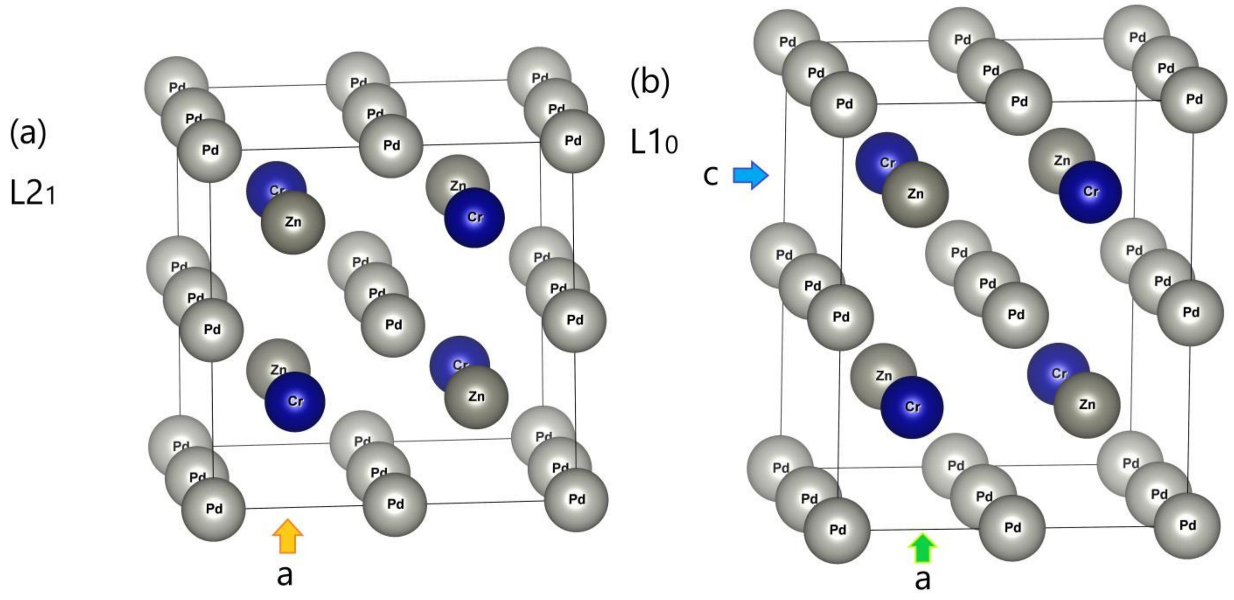

Fig. S3. Crystal structures of cubic $L2_1$-type (a) and tetragonal $L1_0$-type (b) Pd_2CrZn.

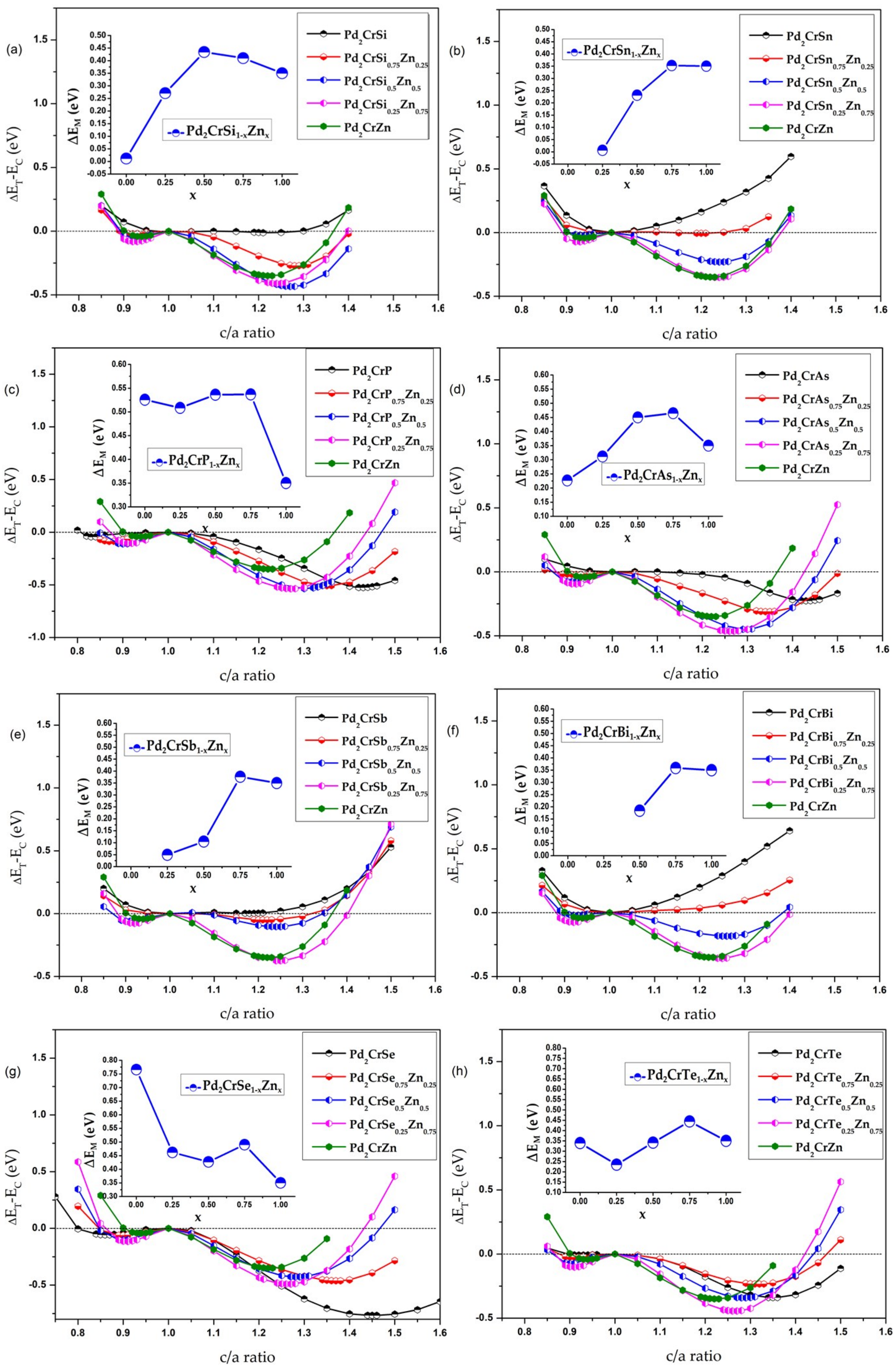

Fig. S4. The total energy differences between tetragonal and cubic states as functions of the c/a ratio of different amount of Zn doping in Pd_2CrZ (Z = Si, Sn, P, As, Sb, Bi, Se, and Te). The zero point of the total energy was set as the energy of the cubic states (c/a = 1).

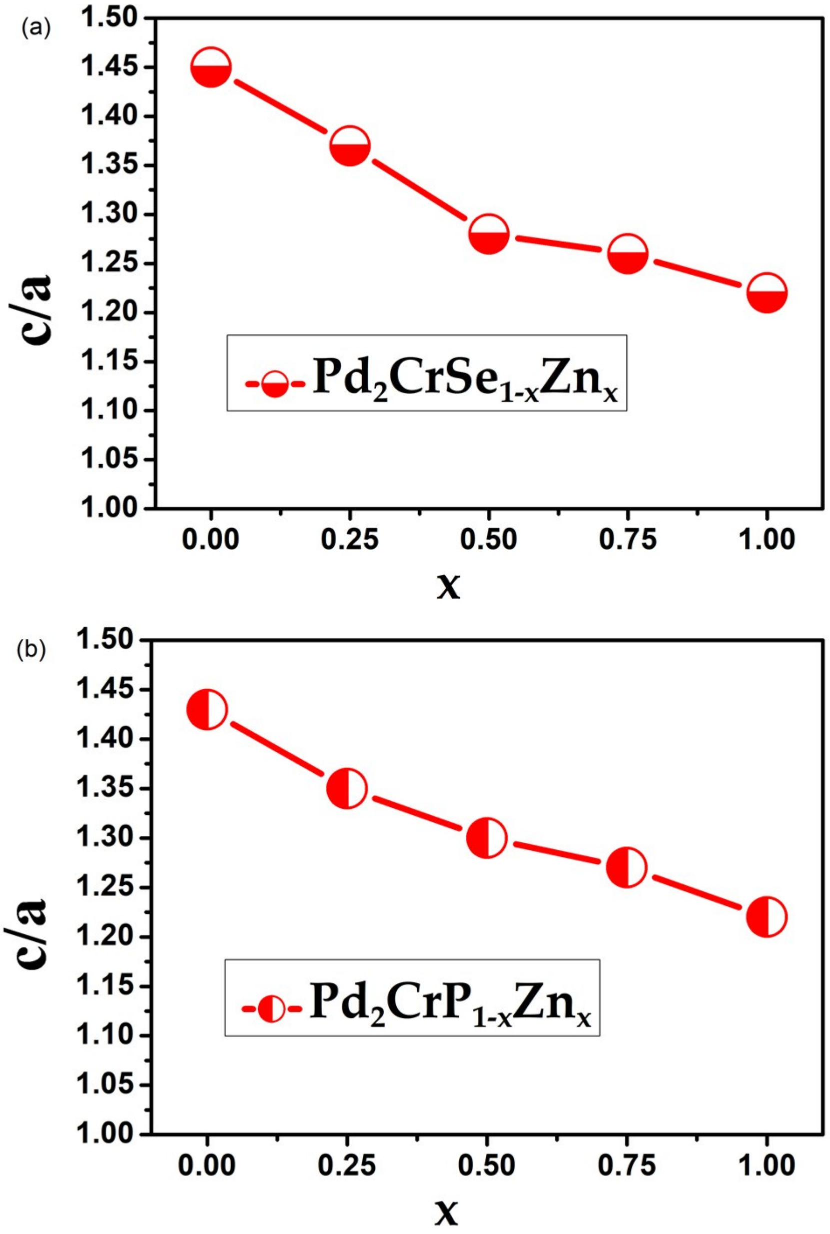

Fig. S5. The c/a ratios as functions of the amount of Zn doping for Pd_2CrZ (Z = Se, P).

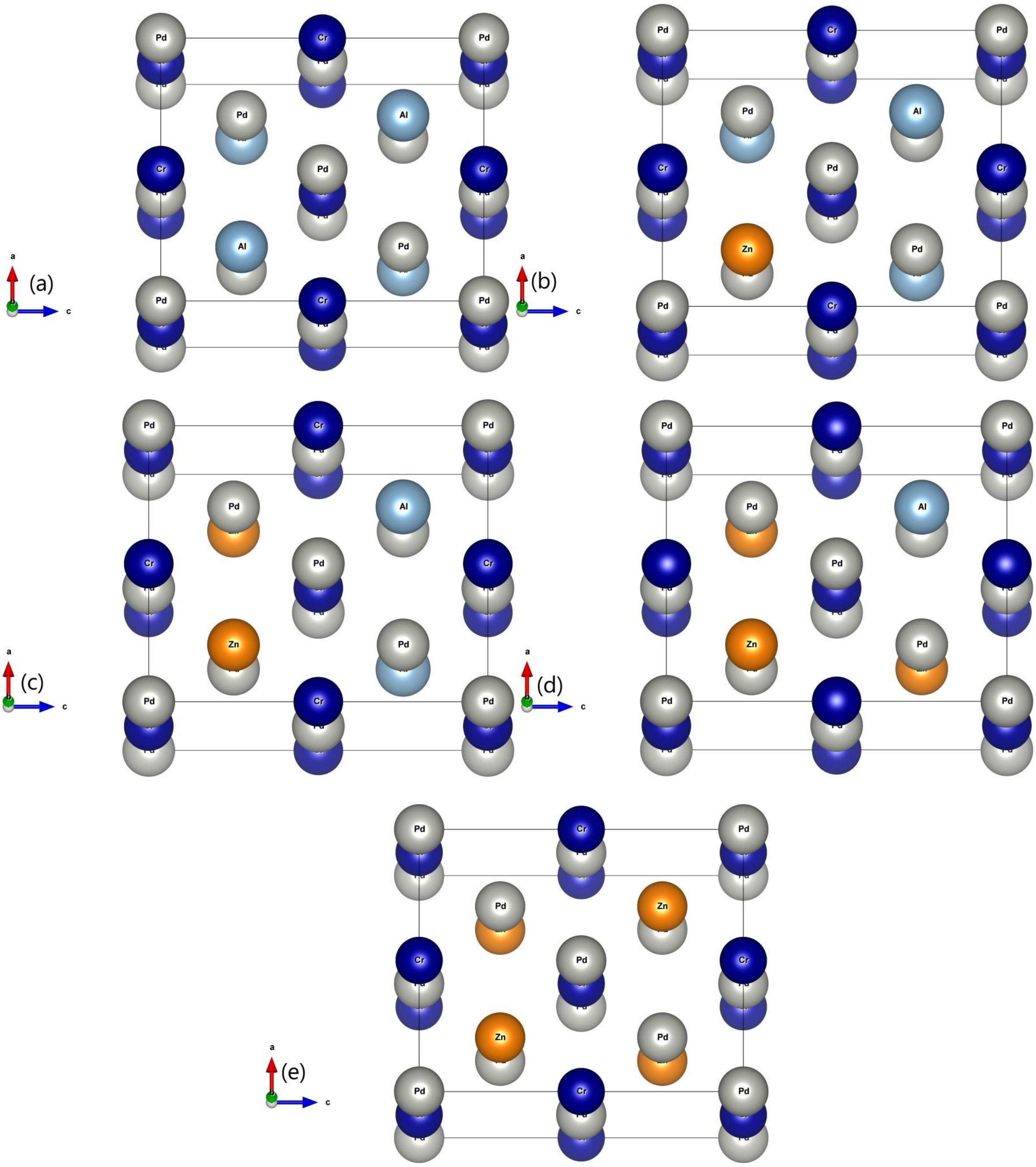

Fig. S6. Crystal structure of $L1_0$ type $Pd_2CrAl_xZn_{1-x}$ (x = 0, 0.25, 0.5, 0.75, 1).
